# Carcinogenic and non-carcinogenic health hazards of potentially toxic elements in commonly consumed rice cultivars in Dhaka city, Bangladesh

**DOI:** 10.1371/journal.pone.0303305

**Published:** 2024-05-14

**Authors:** Nazma Shaheen, Towhid Hasan, Marjia Sultana, Kazi Turjaun Akhter, Ishrat Nourin Khan, Nafis Md. Irfan, Md. Kawser Ahmed

**Affiliations:** 1 Institute of Nutrition and Food Science, University of Dhaka, Dhaka, Bangladesh; 2 Department of Food Technology and Nutrition Science, Noakhali Science and Technology University, Noakhali, Bangladesh; 3 Interdisciplinary Graduate Program in Human Toxicology, University of Iowa, Iowa, Iowa, United States of America; 4 Department of Internal Medicine, University of Iowa, Iowa, Iowa, United States of America; 5 Department of Oceanography, University of Dhaka, Dhaka, Bangladesh; CSIR-Institute of Himalayan Bioresource Technology, INDIA

## Abstract

The study aimed to assess the level of potentially toxic elements (As, Cd, Pb, Zn, Cu, Cr, Mn, and Ni) and associated health implications through commonly consumed rice cultivars of Bangladesh available in Capital city, Dhaka. The range of As, Cd, Pb, Zn, Cu, Cr, Mn, and Ni in rice grains were 0.04–0.35, 0.01–0.15, 0.01–1.18, 10.74–34.35, 1.98–13.42, 0.18–1.43, 2.51–22.08, and 0.21–5.96 mg/kg fresh weight (FW), respectively. The principal component analysis (PCA) identified substantial anthropogenic activities to be responsible for these elements in rice grains. The estimated daily intake (EDI) of the elements was below the maximum tolerable daily intake (MTDI) level. The hazard index (HI) was above the threshold level, stating non-carcinogenic health hazards from consuming these rice cultivars. The mean target cancer risk (TCR) of As and Pb exceeded the USEPA acceptable level (10^−6^), revealing carcinogenic health risks from the rice grains.

## Introduction

The occurrence of potentially toxic elements such as arsenic (As), lead (Pb), chromium (Cr), copper (Cu), cadmium (Cd), zinc (Zn), manganese (Mn), mercury (Hg), nickel (Ni), etc. in the foods can be of geogenic or anthropogenic origins and humans are exposed to them through various pathways [1,2]. Additionally, the natural sources of these elements in the soil are usually dependent on geochemical and geophysical processes (e.g., climate, atmospheric deposition, the nature of the soil, etc.). However, past experience has shown that wastewater irrigation, solid waste disposal, mining, smelting, sewage sludge applications, vehicular exhaust, fertilizers, fungicides, and industrial disposals are the major anthropogenic activities that contribute significantly to the content of potentially toxic elements to the cultivable lands [3].

Agricultural soils can act as a sink for the potentially toxic elements. Cultivation on contaminated soils can lead to elevated toxic elements uptake by crops, and thus affect food quality and safety [4]. Some elements (e.g., Cu, Zn, Cr, and Mn) are essential nutrients for plant growth and/or human and animal nutrition but might be toxic at elevated concentrations. Other metals, such as As, Cd, Hg, Ni, and Pb, are considered potentially toxic and pose health risks to humans and animals at low concentrations due to long-term consumption. These toxic elements may quickly get into the foods through crop uptake, causing toxic health effects to humans [5].

Cereals are widely cultivated in most of the countries of the world. For centuries, cereals have been considered the staple foods in the human diet due to their good-keeping qualities and availability of wide varieties [6]. In many countries, particularly in Southeast Asia, such as Bangladesh, India, Thailand, China, and Vietnam, rice (*Oryza sativa*) is consumed as a staple food. The people of these countries consume rice daily, providing around 70% of their total daily calorie intake [7]. In Bangladesh, more than 80% of the residents keep rice items in their meals even thrice a day, and rice is a vital source of nutrients for poor people who cannot afford diversified foods. Above 80 rice genotypes are cultivated in Bangladesh in *aus*, *aman*, and *boro* seasons with a yearly production of 34.7 million metric tons [8]. Thus, it is reasonable to hypothesize that rice containing potentially toxic elements might cause probable human health hazards beyond its capabilities to meet nutritional requirements as a significant food.

For the last few decades, potentially toxic elements in cereals, especially rice, have been a global issue. Furthermore, the heavy metal pollution in rice may negatively impact the health of rice consumers. Increased levels of As and other potentially toxic metals in the cultivable soils and irrigation water and their uptake by the food crops have been reported [9]. Therefore, there exist few pieces of research on the content of potentially toxic elements and associated health hazards in rice grain collected from different regions of Bangladesh, for example, Chottogram and Mymensingh city [10], Tangail district [8,11], Gaibandha, Natore, Netrokona, Dhaka, Jessore, Jhalakati and Chittagong district [12], Dinajpur district [13], Comilla and Chandpur district [14], Jhenaidah and Kushtia districts [15] and so on. However, to our knowledge, no studies have been conducted yet on the content of potentially toxic elements and associated health hazards in the commonly consumed rice cultivars collected from major markets in Dhaka city, Bangladesh.

Dhaka is the capital of Bangladesh which is also recognized as an overpopulated city [16]. Furthermore, the studied region is a central hub of economic activities where people from all over the country come to live together for better employment, administrative, telecommunication, transport and infrastructure facilities [17]. Being a mega city and a focal point of a country, crops from the other regions of Bangladesh, including the Dhaka region, are sold in the wholesale and retail markets of Dhaka city. Thus, the rice cultivars picked from the diverse markets of Dhaka city could be a representative sample of overall Bangladeshi rice as supplied from different rice-producing regions of the country [18]. But no studies still reported the heavy metal content and its potential health risks in the commonly consumed rice varieties collected from the markets in Dhaka city, Bangladesh. It is utmost important to monitor the heavy metal contamination in the rice varieties to ensure the food safety of a large segment of population. Hence, this study was carried out to investigate the contents of potentially toxic elements (As, Cd, Pb, Zn, Cu, Cr, Mn, and Ni) in commonly consumed rice cultivars collected from various markets of Dhaka City, Bangladesh. This study also assessed the probable carcinogenic and non-carcinogenic health hazards for the adult population consuming analyzed rice cultivars.

## Materials and methods

### Materials

SUPRAPUR^®^ nitric acid (HNO_3_, 69% (v/v)) and hydrogen peroxide (H_2_O_2_) were acquired from Kanto Chemical Co., Japan, and Wako Chemical Co., Japan, respectively. Standard stock solutions of arsenic (As), lead (Pb), copper (Cu), zinc (Zn), chromium (Cr), cadmium (Cd), manganese (Mn), and nickel (Ni) and internal standard solutions of indium (In), cobalt (Co), yttrium (Y), telium (Te), beryllium (Be) and titanium (Ti) were bought from SPEX CertiPrep (Metuchen, NJ, USA). A multi-element solution was purchased from Merck (Darmstadt, Germany). It was used to make a tuning solution with a few elements like indium, barium, lithium, and uranium that can cover a broad range of masses. Ultrapure grade carrier (Argon (Ar)) was received from Air Liquide (Japan). Other chemicals were of analytical grade and purchased from Sigma-Aldrich (St. Louis, USA).

### Sample collection and preparation

More than 80 varieties of rice are cultivated in Bangladesh throughout the year [19]. Based on our market search of four large wholesale markets (Karwan Bazar, Jatrabari Bazar, Shyam Bazar, and Amin Bazar) in Dhaka City, ten varieties were selected that were largely available in the markets and commonly consumed by the residents. The ten most commonly consumed rice cultivars, including Bashful, Katari, Lalbiroi, Basmati, Kaligira, BRRI-32, Naigarsail, Minikit, Pajam, and Chinigura were purchased freshly from the aforementioned four markets. The rice cultivar Minikit is widely available in Bangladeshi markets; however, there is no rice cultivated under this name. This rice is developed by millers through processing hybrid rice such as BRRI-28, BRRI-29, etc.

The studied ten varieties are grown more or less throughout the country except in the southeastern hilly areas and distributed throughout the whole country, including Dhaka city. Hence, the geographic distribution of these selected varieties could not be assessed. To prepare a composite sample for each of the rice cultivars, a food processor was used for homogenization. About 50 g of each rice cultivar was stored at -20 °C in a plastic zip-lock bag to avoid any external contamination for further analysis.

### Analysis of potentially toxic elements

Rice samples were analyzed following the methodology of Shaheen et al. [20] with slight modification. About 5 mL of 69% nitric acid (HNO_3_) and 2 mL of 30% hydrogen peroxide (H_2_O_2_) were added with 0.3 g of the preprocessed samples in a Teflon vessel and digested using a microwave digestion system (Berghof Microwave MWS-2, Germany). After digestion, samples were transferred into a teflon beaker, and total volume was made up to 25 mL with Milli-Q water. The digested solution was then filtered with a 0.45 nm syringe filter and stored in a screw cap plastic tube. The potentially toxic elements presented in the digested samples have been then determined by Inductively coupled plasma mass spectrometer (ICP-MS) (Agilent 7700 series, USA).

### Assurance and control of quality

Standard stock solutions of each potentially toxic element (As, Cd, Pb, Zn, Cu, Cr, Mn, and Ni) were diluted with 69% HNO_3_ to prepare working standard solutions. Calibration curves were developed and the calibration curves with R^2^>0.999 were accepted for concentration calculation. Before starting the sequence, relative standard deviation (RSD<5%) was checked by using a tuning solution. A multielement solution of 1.0 mg/L was used as a tuning solution to cover a wide range of masses of elements. Internal calibration standard solutions containing 1.0 mg/L of indium, yttrium, beryllium, tellurium, cobalt and thallium were added to the digested samples. All test batches were evaluated using an internal quality approach and validated if they satisfied the defined internal quality controls. For each experiment, a run included blank, certified reference materials (CRMs) as an internal standard in samples and samples were analyzed in duplicate to eliminate any batch-specific faults. The CRM (DORM-2–dogfish muscle) procured from the National Research Council (Canada) was analyzed to confirm the analytical performance and good precision (RSD<20%) of the applied method (S1 Table). A detailed quality control and assurance parameters are presented in S1 and S2 Tables.

### Assessment of estimated daily intake (EDI)

The EDIs of the potentially toxic elements were calculated using the following equation [21]:

$$\text{EDI}=\left({\text{E}}_{\text{f}}\times {\text{E}}_{\text{D}}\times {\text{F}}_{\text{IR}}\times {\text{C}}_{\text{M}}\right)/\left({\text{B}}_{\text{W}}\times {\text{T}}_{\text{A}}\right)\;\times \;10^{-3}$$

where E_f_ is exposure frequency (365 days/year), E_D_ is the exposure duration (72.3 years, the average life span for Bangladeshi [22]), F_IR_ is the average food (rice) consumption (328.9 g/person/day [23]), C_M_ is the metal concentration (mg/kg fresh weight (fw)), B_W_ is the average body weight for an adult (60 kg), T_A_ is the average exposure time (365 days × number of exposure years, i.e., 72.3 years in this case).

### Estimation of non-carcinogenic risk

#### Target hazard quotient (THQ)

The THQ was assessed following the equation of Kormoker et al. [15]:

THQ=EDI/RfD

where EDI is the estimated daily metal intake of the population (mg/kg/day) and RfD is the oral reference dose (mg/kg/day). RfDs were found to be 0.0003, 0.001, 0.0035, 0.3, 0.04, 1.5, 0.14 and 0.02 mg/kg body weight/day for As, Cd, Pb, Zn, Cu, Cr, Mn, and Ni, respectively [24].

#### Hazard index (HI)

The HI usually determine the combined potential health risk (non-carcinogenic) of the consumers while multiple heavy metals/potentially toxic elements are consumed from a foodstuff [10]. The HI is the total THQ of the studied metals that was calculated by summing up each of the individual metal’s THQ value for each rice cultivar [10]:

$$\text{HI}\;\left(\text{individual}\;\text{foodstuff}\right)=\text{THQ}\;\left(\text{toxicant}\;1\right)+\text{THQ}\;\left(\text{toxicant}\;2\right)+\ldots +\text{THQ}\;\left(\text{toxicant}\;\text{n}\right)$$

When HI is < 1, no significant health impact is considered. However, HI value > 1 suggests non-carcinogenic health risks may occur due to metals considered [8].

### Determination of carcinogenic risk

The target cancer risk has been computed using the formula from Chakraborty et al. [21] as follows:

TCR=EDI×CSFo

where TCR is the target cancer risk over a lifetime, EDI is the estimated daily metal intake of the population (mg/kg/day) and CSFo is the oral cancer slope factor in (mg/kg/day)^-1^. The CSFo values for As and Pb has been taken as 1.5 (mg/kg/day)^-1^ and 8.5 × 10^−3^ (mg/kg/day)^-1^, respectively from the Integrated Risk Information System database [25].

### Statistical analysis

Data analysis was conducted using MS Excel 2021 (Microsoft Inc., Redmond, USA) and SPSS (v26.0) for Windows (SPSS Inc., Chicago, IL, USA). The concentration of potentially toxic elements in rice grains were presented as mean and standard deviations (SD). Differences among rice cultivars were assessed using a One-way ANOVA analysis. Association among metals in rice cultivars were determined by a Pearson correlation. A Principal component analysis (PCA) was applied to identify the potential sources of heavy metals in the rice cultivars. In addition, the Ward-algorithmic method was used for cluster analysis (CA) with a dendrogram to assess a detailed insight into the distribution of the potentially toxic elements in the rice grains. Statistical significance was set at p < 0.05 for all tests.

## Results and discussion

### Potentially toxic elements in rice verities

The content of potentially toxic elements (As, Pb, Cu, Cr, Mn, Cd, Ni and Zn,) in the ten commonly consumed rice cultivars in Dhaka city, Bangladesh, was presented in Tables 1 and S3. Arsenic (As) is one of the most important global environmental toxicants. It is a ubiquitous element in the environment and has been associated with skin, lung, and bladder cancer. Clinical manifestations of chronic As poisoning include non-cancer endpoint of hyper- and hypo-pigmentation, keratosis, hypertension, cardiovascular diseases and diabetes [26]. Although the groundwater contamination by As is already considered as a serious and severe global environmental problem, but little is known about diet as an additional source of arsenic exposure. The groundwater As contamination in Bangladesh and its further spreading into soil and food crops is in a critical condition [27]. The average As concentration in the rice cultivars was observed to be about 0.18±0.003 mg/kg and followed an ascending order of Chinigura <Bashful <Lalbiroi <Minikit <BRRI-32 <Naigarsail <Katari <Basmati <Pajam <Kaligira (Table 1). The As content was observed above the maximum allowable concentration (MAC) of 0.2 mg/kg recommended by the FAO/WHO for the five rice cultivars (Katari, Basmati, Kaligira, Naigarsail, and Pajam) of this study, suggesting a possible threat of rice contamination by As [28]. The finding of the As content of this study is in line with the previous study conducted by Rahman and Naidu [14], where 0.18 mg/kg of As was reported in Bangladeshi rice. A study by Hasan et al. [19] found As level of about 0.03–0.08 mg/kg in rice samples collected from three major industrial areas of Bangladesh (Savar, Gazipur and Ashulia), which is comparatively lower than this study. Besides, Islam et al. [12] and Kormoker et al. [8] observed relatively higher As content of 0.39–1.0 mg/kg and 0.96–4.66 mg/kg, respectively, in the rice cultivars collected from different districts of Bangladesh compared to the present study.

**Table 1:** Concentration (mg/kg fw) of potentially toxic elements in the rice cultivars.

Rice cultivars	Heavy metal concentration (mg/kg fw)							
	As	Cd	Pb	Zn	Cu	Cr	Mn	Ni
**Bashful**	0.05±0.003^ab^	0.05±0.01^bce^	0.04±0.003^a^	10.74±0.2^ae^	6.99±0.85^abcef^	0.91±0.08^acde^	6.99±0.65^abc^	5.96±1.0^c^
**Katari**	0.21±0.08^abcd^	0.09±0.01^abde^	1.08±0.16^b^	14.49±1.32^abe^	4.93±0.71^bcdef^	0.89±0.02^cde^	4.65±0.58^ac^	0.91±0.09^b^
**Lalbiroi**	0.12±0.04^abd^	0.009±0.002^c^	0.65±0.20^c^	16.63±4.64^abde^	1.98±0.52^de^	0.18±0.06^b^	12.02±1.48^b^	0.21±0.15^b^
**Basmati**	0.25±0.08^abc^	0.13±0.03^ade^	0.15±0.03^a^	18.33±2.11^bde^	9.89±2.42^ae^	1.42±0.38^a^	4.26±1.08^ac^	2.37±0.79^a^
**Kaligira**	0.35±0.04^c^	0.05±0.01^abce^	0.01±0.003^a^	15.47±1.22^abe^	13.42±2.24^g^	1.43±0.31^a^	9.27±1.82^ab^	1.51±0.21^ab^
**BRRI-32**	0.18±0.12^abcd^	0.05±0.01^bce^	0.07±0.01^a^	34.35±1.01^f^	4.79±0.65^bcdf^	0.78±0.06^cde^	22.08±5.03^d^	0.61±0.11^b^
**Naigarsail**	0.21±0.02^abcd^	0.02±0.01^bc^	0.07±0.03^a^	19.83±1.83^bcd^	8.02±0.75^abef^	0.72±0.11^cd^	11.37±0.03^b^	1.08±0.42^ab^
**Minikit**	0.17±0.12^abcd^	0.15±0.06^de^	0.16±0.14^a^	26.23±3.64^cd^	4.35±0.49^cdf^	0.65±0.04^bcd^	2.54±0.09^c^	1.41±0.49^ab^
**Pajam**	0.27±0.08^bcd^	0.04±0.04^bce^	0.09±0.001^a^	22.22±1.59^d^	8.29±0.1^aef^	0.84±0.15^de^	5.31±0.96^c^	0.59±0.18^b^
**Chinigura**	0.04±0.002^bc^	0.1±0.004^ade^	0.06±0.004^a^	12.35±0.03^ae^	6.09±0.48^bcf^	1.26±0.17^ae^	2.51±0.16^c^	0.51±0.02^b^
**MAC**	0.2 [28]	0.4 [28]	0.2 [28]	NA	20 [29]	1.0 [29]	NA	0.5 [29]

fw: Fresh weight, MAC: Maximum allowable concentration. NA: Not applicable. Different letters within same column are significantly different (p<0.05).

Cd, a metallic element, occurs naturally at low levels in the environment and is toxic even at extremely low levels. Like air and water, foods also serve as the prime source of cadmium exposure. In humans, long-term exposure to Cd may result in renal dysfunction, bone defects, osteomalacia, osteoporosis and spontaneous fractures, increased blood pressure, etc. [30]. This study observed the average Cd concentration in the rice cultivars about 0.07±0.005 mg/kg. The concentration of Cd in all the rice cultivars was found below the MAC of 0.4 mg/kg by FAO/WHO [28]. The concentration of Cd in rice cultivars increases following the ascending order of Lalbiroi <Naigarsail <Pajam <BRRI-32 <Bashful <Kaligira <Katari <Chinigura <Basmati <Minikit (Table 1). The previous studies observed the Cd content in Bangladeshi rice at about 0.75±0.23 and 0.093±0.016 mg/kg in the study of Kormoker et al. [15] and Proshad et al. [11], respectively, which are within the range of Cd concentration of this study. Islam et al. [12] observed 0.05±0.02 mg/kg of Cd content in Naigarsail rice, which is similar to this study and 0.21±0.22 mg/kg of Cd content in Basmati rice, which is higher than the present study.

Pb, the most significant potentially toxic elements, is absorbed in inorganic forms through ingestion by food, water, and inhalation. A severe effect of Pb toxicity is its teratogenic effect. Also, Pb poisoning might cause the inhibition of hemoglobin synthesis, dysfunctions in the urinary, reproductive and cardiovascular systems, and acute and chronic damage to the gastrointestinal tract, urinary tract and so on. Pb toxicity in children could lead to poor brain development, resulting in poor intelligence outcomes [31]. The average Pb concentration in the rice cultivars was found 0.23±0.003 mg/kg (0.01–1.08 mg/kg) and followed the ascending order of: Pajam <Kaligira <Bashful <Chinigura <Naigarsail <BRRI-32 <Basmati <Minikit <Lalbiroi <Katari (Table 1). The Pb concentration of all the rice cultivars except Lalbiroi and Katari were observed below the MAC (0.2 mg/kg) [28]. The findings of this study showed congruity with the previous study conducted by Islam et al. [12], where Pb concentration in the range of 0.90–1.12 mg/kg in different cultivars of Bangladeshi rice (Basmati, Kaligira, Naigarsail, Minikit, BRRI-32, Red Biroi) collected from seven districts (Gaibandha, Netrokona, Natore, Jossore, Dhaka, Chottogram, Jhalokati) in Bangladesh were observed. However, a previous study by Hasan et al. [19] reported a higher Pb content of about 1.3 mg/kg in the rice samples collected from Savar and Ashulia City, Bangladesh, than the present study.

Zn is considered relatively non-toxic, especially if taken orally, but excess amount can cause system dysfunctions resulting in impairment of growth and reproduction. The clinical signs of Zn toxicosis have been reported as vomiting, diarrhea, bloody urine, icterus (yellow mucous membrane), liver failure, kidney failure, and anemia [32]. This study observed the Zn content in the range of 10.74±0.202 (Bashful) to 34.35±1.013 (BRRI-320) mg/kg with the following ascending order of Bashful <Chinigura <Katari <Kaligira <Lalbiroi <Basmati <Naigarsail <Pajam <Minikit <BRRI-32 in the Bangladeshi rice cultivars (Table 1). The finding is in line with the study of Islam et al. [12], where Zn concentration was observed in the range of 7.52±1.14–13.98±7.50 mg/kg in the rice cultivars (Basmati, Kaligira, Naigarsail, Minikit, BRRI-32, Red Biroi) collected from different districts of Bangladesh. However, Hossen et al. [13] and Hasan et al. [19] found higher Zn concentration in the Bangladeshi rice samples compared to the present study, which was about > 43 mg/kg and > 97 mg/kg, respectively.

Cu is one of the essential micronutrients, however, in excess consumption, it may cause untoward health effects like liver and kidney damage [32]. The mean Cu content of rice cultivars analyzed in this study followed the ascending order of Lalbiroi <Minikit <BRRI-32 <Katari <Chinigura <Bashful <Naigarsail <Pajam <Basmati <Kaligira (Table 1). This study observed Cu concentration of 6.876±0.847 mg/kg (1.98±0.52 mg/kg in Lalbiroi to 13.42±2.24 mg/kg in Kaligira) in the rice cultivars (Table 1). None of the rice cultivars were found above the recommended MAC of 20 mg/kg by FAO/WHO [29]. The findings of Cu content in rice cultivars are in line with the previous study conducted by Islam et al. [12] and Hossen et al. [13], reporting about 11–15 mg/kg and 13–15 mg/kg of Cu content in the Bangladeshi rice, respectively, but lower than the study by Hasan et al. [19] reporting 19–22 mg/kg of Cu content in the rice samples.

Cr toxicity might have few adverse effects on human health, especially on the human immune system [33]. In this study, the average Cr content in the rice cultivars has been observed as 0.91±0.40 mg/kg (0.18±0.06 mg/kg in Lalbiroi to 1.43±0.31 mg/kg in Kaligira) in the ascending order of Kaligira <Basmati <Chinigura <Bashful <Katari <Pajam <BRRI-32 <Naigarsail <Minikit <Lalbiroi (Table 1). All of the rice cultivars in this study (except Basmati, Kaligira and Chinigura) have a Cr concentration below the MAC of 1.0 mg/kg [29]. Zakir et al. [34] observed higher Cr content (10–15 mg/kg) in the rice cultivars obtained from the rice fields of Bhaluka area, Mymensingh, where industrial wastewater was used for irrigation. The differences from the findings of this study could be due to their use of industrial wastewater for irrigation that may have high Cr content.

Manganese can be found in nature in their oxidation states, and subsequently, the manganese oxides are released into the environment. Although it has several physiological functions, but excessive consumption may causes substantial toxicity, such as neurotoxicity, hepatotoxicity, nephrotoxicity, and so on [33]. The lowest and highest Mn contents were found in Chinigura (2.51±0.16 mg/kg) and Lalbiroi (12.02±1.48 mg/kg), respectively, with the mean Mn concentration of 8.09±0.66 mg/kg in the rice cultivars (Table 1). The mean Mn content of the studied rice cultivars followed the ascending order of BRRI-32 <Lalbiroi <Naigarsail <Kaligira <Bashful <Pajam <Katari <Basmati <Minikit <Chinigura (Table 1). The result is supported by a previous work by Islam et al. [12] reporting Mn content in the range of 8–19 mg/kg in rice cultivars obtained from various markets in different districts in Bangladesh, while Hossen et al. [13] found higher concentration of Mn (> 20 mg/kg) in rice cultivars (Boro, Aus, Aman) collected from Dinajpur district, Bangladesh.

Typically, a very tiny amount of Nickel (Ni) can be found in the environment. Still, it can be attributed to a wide range of pulmonary diseases, including fibrosis, tumors, lung inflammation and emphysema [31]. The mean Ni content in the rice cultivars was observed 1.52±1.00 mg/kg, with Lalbiroi having the lowest (0.21±0.15 mg/kg) and Bashful having the highest Ni content (5.96±1.00 mg/kg) (Table 1). The analysed rice cultivars in this study followed the ascending order of Bashful <Basmati <Kaligira <Minikit <Naigarsail <Katari <BRRI-32 <Pajam <Chinigura <Lalbiroi (Table 1). Among the studied cultivars, all the rice cultivars except Lalbiroi contained lower MAC than the recommended value of 0.5 mg/kg [29]. A previous study by Kormoker et al. [8] found a similar Ni level in rice cultivars that is about 3.8–13 mg/kg collected from the fields around industrial areas of Tangail district, Bangladesh. On the other hand, a lower Ni level of 0.18 mg/kg was observed in rice samples collected from Gazipur, Bangladesh in the study of Hasan et al. [19].

It is worth noting that a comparison of potentially toxic element levels in rice samples in this study to that of previous studies in Bangladesh is quite difficult. There are a few factors that might affect the content of potentially toxic elements in rice, such as varietal genetic features in rice, absorption and accumulation capabilities of rice grain, soil properties, types of fertilizers and pesticides used, climatic conditions, sources of irrigation water, etc. [15]. The above factors would vary among the studies conducted in different geographical locations [35], which might be a possible reason behind the dissimilar findings of the content of potentially toxic elements in the rice cultivars found in this study compared to the previous studies. In contrast, our study found similar levels of heavy metals with previous studies in rice samples, indicating that the heavy metals content remained unchanged over time. This may be due to the lack of proper practices in agricultural systems, such as excessive use of chemical fertilizers and/or pesticides for a long time, use of irrigation water contaminated with toxic elements, lack of proper waste management from industries, etc. [32]. Therefore, further research is needed to determine the environmental fate of heavy metals, their transport pathways, and potential toxicity through the food webs to minimize contamination. In addition, synchronization of local studies aligned with current global laws on heavy metal contamination is also essential to improve food safety in Bangladesh [36].

### Sources of potentially toxic metals in rice cultivars

Associations among the concentrations of the potentially toxic elements of the rice cultivars were investigated and presented in Table 2. Among the rice cultivars, a strong correlation (p<0.01) was observed between As and Cu (r = 0.50), Cd and Mn (r = -0.53), Cu and Cr (r = 0.79), and Mn and Zn (r = 0.55). The association between Cd and Cr (r = 0.37), Cu and Pb (r = -0.46) and Zn and Ni (r = -0.38) was also found statistically significant (p<0.05). The higher inter-metal correlations of the toxic elements found in these rice cultivars may reveal the ubiquitous sources or common pathways of metal contamination and/or parallel metal accumulation properties of the rice cultivars [37].

**Table 2:** Pearson’s correlation coefficient matrix for potentially toxic elements in rice cultivars.

	**As**	**Cd**	**Pb**	**Zn**	**Cu**	**Cr**	**Mn**	**Ni**
As	1.00							
Cd	0.09	1.00						
Pb	-0.007	0.07	1.00					
Zn	0.22	0.05	-0.24	1.00				
Cu	0.50**	0.05	-0.46*	-0.24	1.00			
Cr	0.17	0.37*	-0.32	-0.29	0.79**	1.00		
Mn	0.01	-0.53**	-0.10	0.55**	-0.14	-0.29	1.00	
Ni	-0.22	0.13	-0.23	-0.38*	0.25	0.23	-0.18	1.00

*P˂0.05.

**P˂0.01.

**Fig 1:**
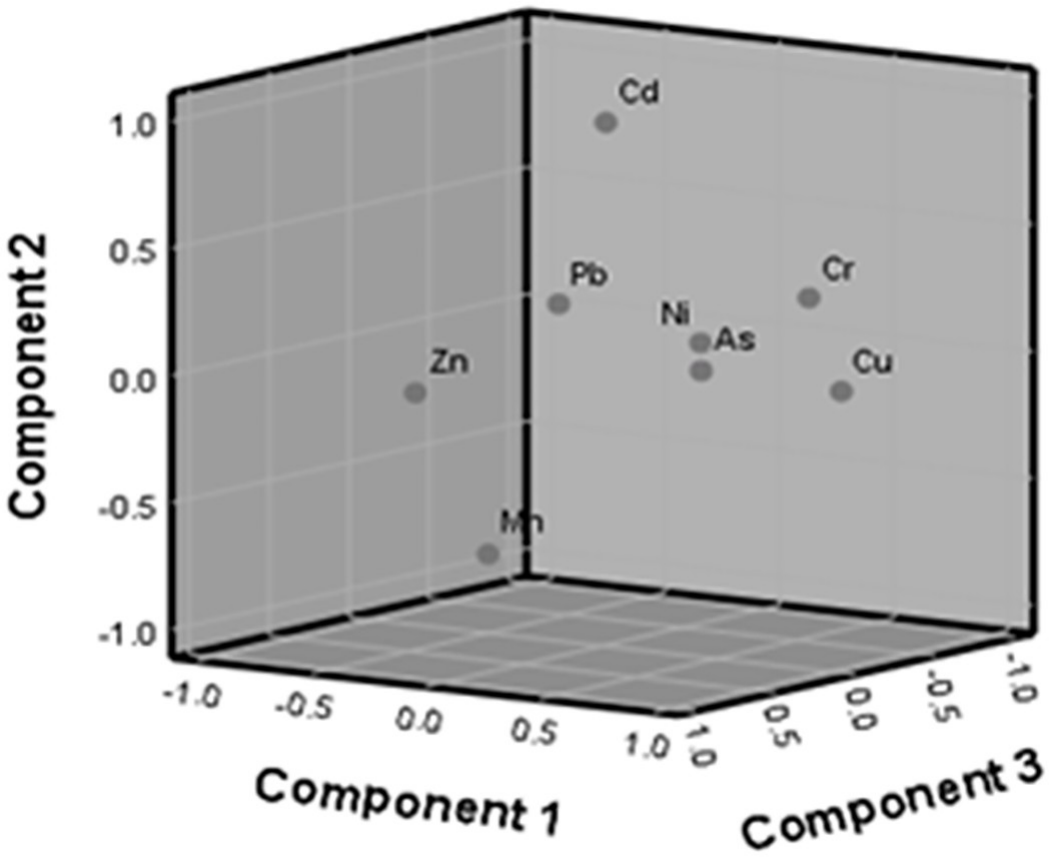
Principal component analysis loading plots for the three components.

To identify the sources of potentially toxic elements, principal component analysis (PCA) is the most widely employed tool [37]. It was also applied in the present study (Fig 1 and Table 3), and the principal components with eigenvalues higher than 1 (before and after rotation) were extracted. The results revealed a total of four components having eigenvalues higher than one. The first principal component (PC1) consisted of Cd, Cu, Cr, and Ni, accounting for 32.44% of the total variance. The As, Zn, Cu, and Mn were observed in the second components (PC2), which explains 22.51% of the total variance. The third component (PC3) described 16.82% of the total variance that was strongly correlated with As, Cd, and Pb. The fourth component (PC4) loaded heavily on Cd and Zn, explaining 12.73% of all the variance. The principal components containing the potentially toxic elements in rice cultivars might result from a mixture of anthropogenic sources (industrial afferents and agricultural practices) as the rice samples were collected from wholesale markets in the capital city of Bangladesh, where crops are usually brought from all over the country including urban and rural areas [15]. Overall, PCA resulted in a condensation of the initial dimension of the dataset to four components, which explained 84.50% of the data variation (Table 3).

**Table 3:** Total variance explained and component matrices for the potentially toxic elements in rice cultivars.

**Component**	**Initial eigenvalues**			**Extraction sums of squared loadings**			**Rotation sums of squared loadings**		
	**Total**	**% of Variance**	**Cumulative %**	**Total**	**% of Variance**	**Cumulative %**	**Total**	**% of Variance**	**Cumulative %**
1	2.60	32.44	32.44	2.60	32.44	32.44	2.32	29.04	29.04
2	1.80	22.51	54.94	1.80	22.51	54.95	1.53	19.06	48.10
3	1.35	16.82	71.76	1.35	16.82	71.77	1.52	18.99	67.09
4	1.02	12.73	84.49	1.02	12.73	84.50	1.40	17.40	84.50
5	0.60	7.57	92.05						
6	0.49	6.06	98.12						
7	0.10	1.19	99.31						
8	0.06	0.69	100.00						
**Elements**	**Component matrix**					**Rotated component matrix**			
	**PC1**	**PC2**	**PC3**	**PC4**		**PC1**	**PC2**	**PC3**	**PC4**
As	0.23	0.57	0.56	-0.35		0.48	0.03	0.07	0.76
Cd	0.44	-0.26	0.59	0.59		0.07	0.97	0.08	0.01
Pb	-0.35	-0.57	0.45	-0.42		-0.58	0.09	-0.56	0.41
Zn	-0.49	0.63	0.25	0.51		-0.18	-0.001	0.92	0.29
Cu	0.81	0.49	-0.07	-0.26		0.98	-0.03	-0.10	0.08
Cr	0.87	0.19	0.03	0.01		0.81	0.32	-0.13	-0.09
Mn	-0.58	0.59	-0.33	-0.04		-0.12	-0.69	0.54	0.09
Ni	0.49	-0.27	-0.56	0.21		0.29	0.08	-0.19	-0.73

Extraction method: Principal component analysis; Rotation method: Varimax with Kaiser normalization.

**Fig 2:**
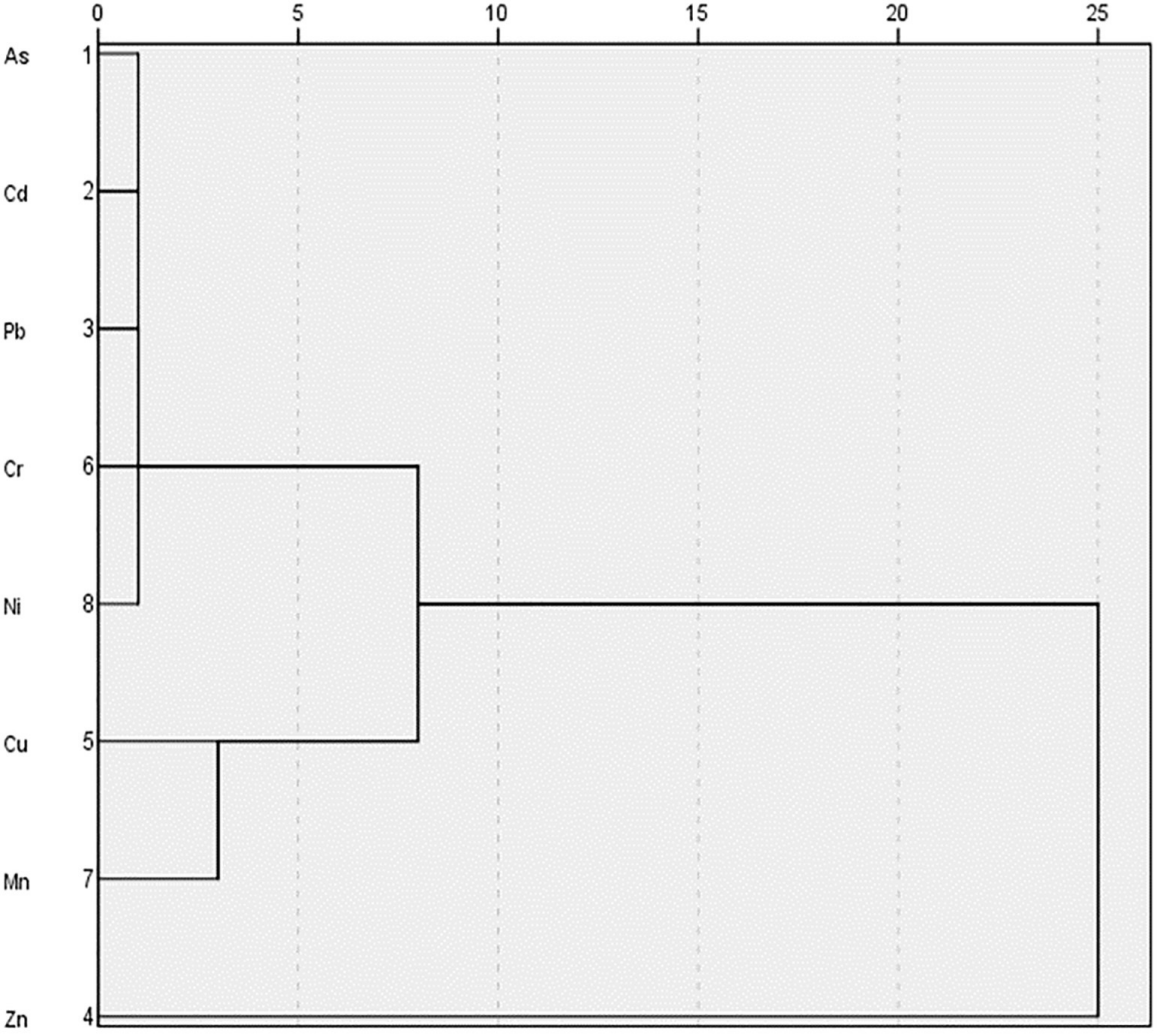
Dendrogram obtained by cluster analysis for heavy metal in rice cultivars.

Moreover, CA was conducted to ascertain the correlation among the analyzed potentially toxic elements and their probable sources. The cluster analysis (CA) provided a dendrogram with several cluster shapes among the studied toxic elements that were presented in Fig 2. The metals attributed in the same cluster were assumed to be resembled in nature. Also, heavy metals in the rice cultivars were grouped into primary clusters, showing strong significant correlations to each other. The primary clusters of As, Cd, Pb, Cr and Ni, and Cu and Mn were formed within a distance of five on the scale (Fig 2). Kormoker et al. [8] also added that As, Cd, Ni, and Pb were included in the same cluster when rice samples were collected from different agricultural fields of Tangail district, Bangladesh and pollution could be attributed to the mixture of the anthropogenic sources. Thus, it can be affirmed that the heavy metal content of the rice cultivars could be due to the emissions of metals in the irrigation water and cultivated soil deriving from industrial activities such as chemical leaching and precipitation, agricultural practices such as the use of pesticide and fertilizer, etc. and further accumulation of the metals by crops [38].

### Estimated daily intake (EDI) of potentially toxic elements

Table 4 shows the EDI of the heavy metals from each of the rice cultivars and compared with the maximum tolerable daily intake (MTDI). The EDI of As, Cd, Pb, Zn, Cu, Cr, Mn, and Ni by an adult has been found about 0.001, 0.0004, 0.001, 0.12, 0.04, 0.006, 0.05 and 0.009 mg/day, respectively from the consumption of the rice cultivars. Results also revealed that the highest and lowest estimated intake of toxic elements varied among the rice cultivars. For instance, the estimated As intake was observed to be the highest and lowest from Kaligira (0.002 mg/day) and Chinigura (0.0002 mg/day) varieties, respectively. Similarly, Cd from Miniket (0.0009 mg/day) and Lalbiroi (5.4E-05 mg/day), Pb from Katari (0.007 mg/day) and Pajam (5.4E-05 mg/day), Zn from BRRI-32 (0.21 mg/day) and Bashful (0.07 mg/day), Cu from Kaligira (0.08 mg/day) and Lalbiroi (0.01 mg/day), Cr from Kaligira (0.009 mg/day) and Lalbiroi (0.001 mg/day), Mn from BRRI-32 (0.14 mg/day) and Chinigura (0.02 mg/day), and Ni from Bashful (0.04 mg/day) and Lalbiroi (0.001 mg/day). The EDIs of these toxic metals from rice cultivars were observed below the corresponding MTDI values. The results of this study are in line with the previous studies conducted by Kormoker et al. [15] in Jhenaidah district and by Proshad et al. [11] in Tangail district. Both of these studies found the EDIs of the toxic elements (Cd, Cu, As, Ni, Pb) from the rice consumption below the MTDI values of the respective metals for the adult population. However, from the above findings assessing the comparison between the EDI and MTDI of the potentially toxic elements, it can be inferred that the ten rice cultivars of this study may not lead to health risks to adult consumers.

**Table 4:** Estimated daily intake (EDI) of potentially toxic elements through consumption of rice cultivars with the corresponding maximum tolerable daily intake (MTDI).

Rice cultivars	Estimated daily intake (EDI) (mg/day)							
	As	Cd	Pb	Zn	Cu	Cr	Mn	Ni
**Bashful**	0.0003^a^	0.0003^a^	0.0002^a^	0.07^a^	0.04^ac^	0.006^ad^	0.04^a^	0.04^a^
**Katari**	0.001^b^	0.0005^b^	0.007^b^	0.09^ab^	0.03^ab^	0.005^a^	0.03^a^	0.006^be^
**Lalbiroi**	0.0007^c^	5.4E-05^a^	0.004^c^	0.10^bd^	0.01^b^	0.001^b^	0.07^b^	0.001^c^
**Basmati**	0.001^b^	0.0008^c^	0.0009^d^	0.11^bd^	0.06^c^	0.009^c^	0.03^a^	0.014^d^
**Kaligira**	0.002^b^	0.0003^a^	0.00008^d^	0.09^ab^	0.08^c^	0.009^c^	0.06^b^	0.009^b^
**BRRI-32**	0.001^b^	0.0003^a^	0.0004^a^	0.21^c^	0.03^ab^	0.005^a^	0.14^c^	0.004^e^
**Naigarsail**	0.001^b^	9.6E-05^a^	0.0004^a^	0.12^d^	0.05^ac^	0.004^a^	0.07^b^	0.007^b^
**Minikit**	0.001^b^	0.0009^c^	0.0009^d^	0.16^e^	0.03^ab^	0.004^a^	0.02^a^	0.009^b^
**Pajam**	0.001^b^	0.0002^a^	5.4E-05^a^	0.14^e^	0.05^ac^	0.005^a^	0.03^a^	0.004^e^
**Chinigura**	0.0002^a^	0.0005^b^	0.0004^a^	0.08^a^	0.04^ac^	0.008^cd^	0.02^a^	0.003^ce^
**MTDI**	0.126 [39]	0.046 [40]	0.21 [41]	60 [42]	30 [43]	0.2 [44]	2–5 [44]	0.3 [42]

MTDI: Maximum tolerable daily intake. Different letters within same column are significantly different (p<0.05).

**Fig 3:**
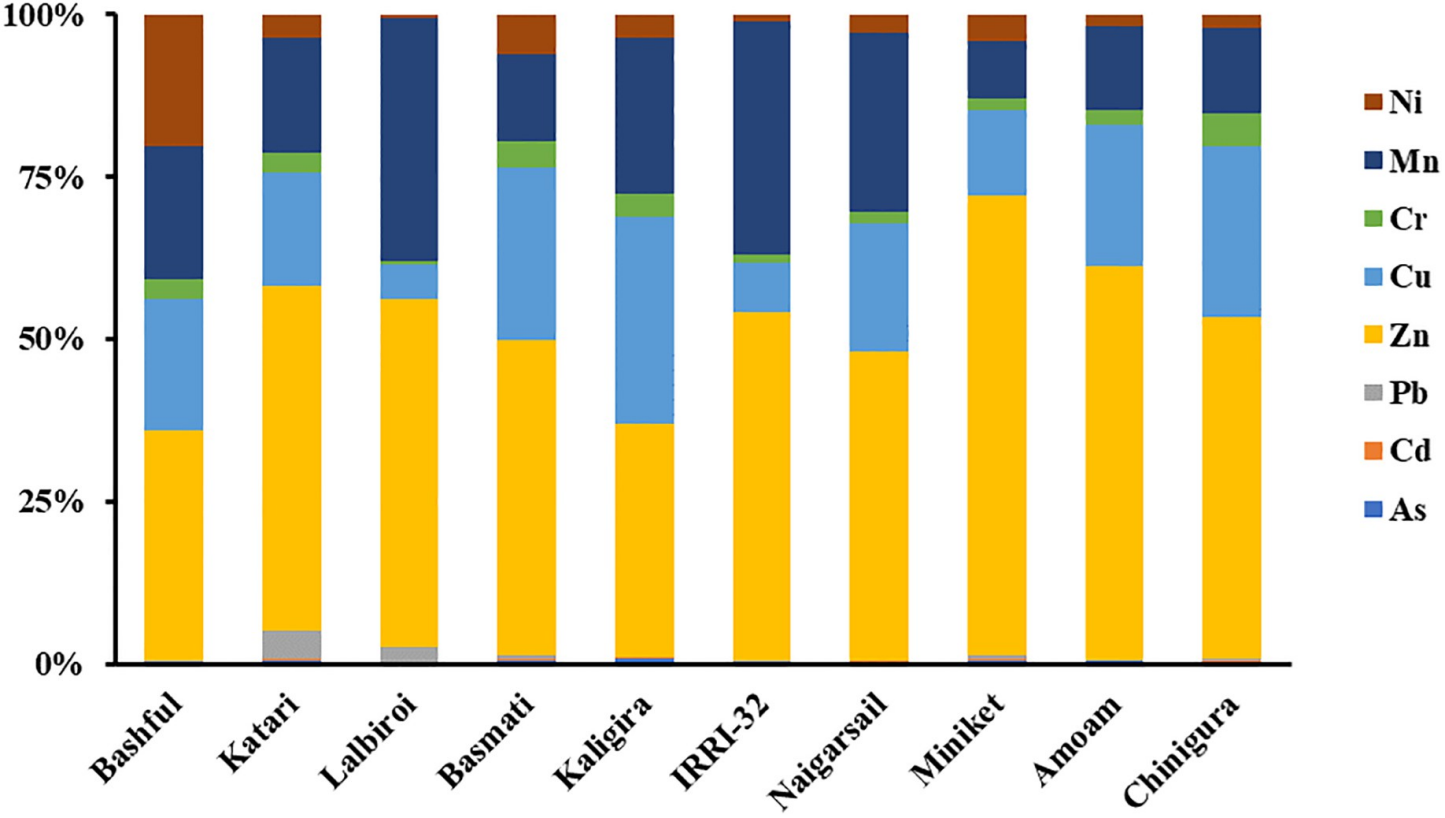
Percentage distribution of different heavy metals among different rice cultivars.

Additionally, for better analysis of the source difference of the toxic elements from rice cultivars, the percent contribution of the elements among rice cultivars has been compared. It is clear from the Fig 3 that the percent distribution of the studied trace metals differed significantly among the rice cultivars. Among the studied metals, Zn was found in the highest percentage in all the rice cultivars, ranging from 35.95% (Kaligira) to 70.86% (Minikit). The contribution to potentially toxic elements among the rice cultivars, varied in the following ascending order of: Lalbiroi <Basmati <Kaligira <Bashful <Minikit <Pajam <Chinigura <Naigarsail <BRRI-32 <Katari that might enter into human body via consumption. Hence, being the staple food, special focus should be given to the content of the studied trace elements in the above rice cultivars, and increased supervision by the government is crucial to minimize the potential health hazards of heavy metals to humans through consumption.

### Health risk assessment

#### Non-carcinogenic risk

The non-carcinogenic health hazards of the adult consumers from the rice cultivars available in the markets of Dhaka city have been evaluated using THQ and shown in Table 5. The THQ is a ratio of the determined dose of a pollutant to a reference dose level. Although the estimation of the THQ method does not provide a quantitative estimate of the probability of an exposed population experiencing a reverse health effect, it indicates the risk level due to the contaminant exposure. The population is considered safe, with a probability of unfavorable effects and unsafe when the THQ value is < 1, between 1 to 5, and > 5, respectively, when they are exposed to potentially toxic elements [10]. Table 5 delineated the THQ of the analyzed trace elements from consuming the above rice cultivars.

**Table 5:** The Target hazard quotients (THQ) (Noncarcinogenic) values for individual metals from consumption of rice cultivars.

Rice cultivars	Target hazard quotients (THQ)								HI
	As	Cd	Pb	Zn	Cu	Cr	Mn	Ni	
**Bashful**	1.06	0.32	0.07	0.22	1.07	0.004	0.31	1.82	4.87
**Katari**	4.32	0.54	1.88	0.30	0.75	0.004	0.20	0.28	8.28
**Lalbiroi**	2.41	0.05	1.14	0.34	0.30	0.001	0.52	0.07	4.84
**Basmati**	5.04	0.78	0.27	0.37	1.51	0.006	0.19	0.73	8.90
**Kaligira**	7.12	0.33	0.02	0.32	2.05	0.006	0.41	0.46	10.71
**BRRI-32**	3.50	0.31	0.12	0.70	0.73	0.003	0.96	0.19	6.52
**Naigarsail**	4.26	0.10	0.12	0.40	1.23	0.003	0.50	0.33	6.94
**Minikit**	3.39	0.91	0.27	0.53	0.67	0.003	0.11	0.43	6.33
**Pajam**	5.55	0.24	0.02	0.45	1.27	0.003	0.23	0.18	7.95
**Chinigura**	0.72	0.59	0.11	0.25	0.93	0.005	0.11	0.16	2.87
**THQ of each metal through rice consumption**	3.74	0.42	0.40	0.39	1.05	0.004	0.35	0.46	6.82

HI: Hazard index.

Table 5 shows that among the observed toxic elements, the average THQ of As and Cu were above the limit of 1, concluding that consumers might experience significant non-carcinogenic health hazards after ingestion of As and Cu from the studied rice cultivars. On the other hand, Cd, Pb, Zn, Cr, Mn and Ni may not cause a non-carcinogenic risk for the adults because of their low average THQ value (less than 1) observed in the studied rice cultivars (Table 5). This finding is slightly different from a previous study by Kormoker et al. [8], who stated that the THQ value for the individual toxic elements (Ni, Cu, As, Cd, Pb) excluding Cr, was > 1, suggesting potential non-carcinogenic health hazards for the adult population from consuming the rice produced in Tangail district, Bangladesh. In addition, the THQ values for the potentially toxic elements (Cu, Zn, Ni, Pb, Mn, Fe) in rice were exceeded 1 for adults in the previous study conducted by Zakir et al. [10], where it is inferred that ingestion of rice found in Mymensingh and Chottogram city may pose non-carcinogenic health risks.

Table 5 also shows that among all the potentially toxic elements of this study, the average HI through consuming the rice cultivars was 6.82 (>1). The highest and lowest HI was noticed through the consumption of Kaligira (10.71) and Chinigura rice (2.87), respectively, for the adult consumers. As was observed as the major risk contributor for the analyzed rice cultivars, accounted for 55% of the HI, while Cu was the second risk contributor (16% of the HI). However, the findings of the noncarcinogenic health risks revealed that the rice cultivars collected from the markets of Dhaka city were not safe for the consumption of the adult population. Thus, people would have substantial non-carcinogenic health threats from consuming these rice cultivars.

#### Carcinogenic risk

As and Pb may cause both non-carcinogenic and carcinogenic health consequences based on their exposed dose. The target cancer risk (TCR) derived from As and Pb was calculated in this study and presented in Table 6. The TCR of As and Pb observed in the range of 3.22E-04 (Chinigura) to 3.20E-03 (Kaligira) and 4.63E-07 (Pajam) to 5.60E-05 (Katari), respectively, in the rice cultivars of this study. According to USEPA, the TCR values above 10^−6^ for As and Pb are usually considered a threshold level for causing cancer in the adult population [25]. In this study, the TCR values of As were observed above the threshold level for all the rice cultivars. In addition, the TCR values of Pb were also found above 10^−6^ for all the rice cultivars except Pajam and Kaligira. The previous studies also observed the TCR of As and Pb above the acceptable level of 10^−6^ for the rice samples consumed in Bangladesh [8,15]. However, the results of this study allude the fact that consumption of different rice cultivars from the markets of Dhaka city would pose carcinogenic health hazards for the consumers.

**Table 6:** Target cancer risk (TCR) As and Pb due to consumption of rice cultivars.

Rice cultivars	Target cancer risk (TR)	
	As*	Pb
**Bashful**	4.75E-04	2.04E-06
**Katari**	1.95E-03	5.60E-05
**Lalbiroi**	1.09E-03	3.40E-05
**Basmati**	2.27E-03	8.01E-06
**Kaligira**	3.20E-03	6.71E-07
**BRRI-32**	1.58E-03	3.54E-06
**Naigarsail**	1.92E-03	3.47E-06
**Minikit**	1.53E-03	8.14E-06
**Pajam**	2.50E-03	4.63E-07
**Chinigura**	3.22E-04	3.28E-06

*Assuming 50% inorganic As in foods [8].

## Conclusion

The concentrations of the potentially toxic elements in the rice cultivars obtained from the four markets of Dhaka city, Bangladesh varied extensively. The average daily intake of the potentially toxic elements from these rice cultivars was below the WHO and FAO permissible levels recommending no unfavorable health effects. Nonetheless, from the standpoint of human health, the THQ values for As (except for Chinigura) and Cu (except for Katari, Lalbiroi, BRRI-32, Minikit) were observed above 1, suggesting considerable health risks for the people if they ingest As and Cu through the analyzed rice cultivars. The findings also revealed that As was the primary hazard contributor for these most frequently consumed rice cultivars, accounting for more than half of the total THQ, followed by Cu (16%). The HI through the consumption of the rice cultivars of this study was observed above 1, which reveals potential non-carcinogenic health risks for adult consumers from consuming the rice cultivars. Moreover, the average TCR values of As and Pb surpassed the accepted risk level of USEPA of 1×10^−6^ (except Pajam and Kaligira for Pb), indicating a threat of cancer induced by As and Pb to the consumers. However, the present study exhibited that the potential health risks associated with consuming these rice cultivars are not negligible, and thus, more attention should be paid to minimizing and controlling the sources of metal pollution to achieve food quality and safety and protect the health and wellbeing of the end user from food contamination.

## Supporting information

S1 Table Measured and certified values of heavy metal concentration (mg/kg) in standard reference material of INCTCF-3 –Corn flour and DORM-2 –Dogfish muscle (National Research Council, Canada). (DOCX)

S2 Table ICP-MS operating conditions and measurement parameters. (DOCX)

S3 Table Heavy metals concentration (mg/kg fw) in rice samples. (DOCX)
