# Supplementary material for: Carcinogenic and non-carcinogenic health hazards of potentially toxic elements in commonly consumed rice cultivars in Dhaka city, Bangladesh
Source: PLoS One. 2024 May 14;19(5):e0303305. doi: 10.1371/journal.pone.0303305 (PMC11093395; doi:10.1371/journal.pone.0303305)
Supplement: S1 Table — (DOCX) [file pone.0303305.s001.docx]

**S1 Table.** Measured and certified values of heavy metal concentration (mg/kg) in standard reference material of INCTCF-3 – Corn flour and DORM-2 – Dogfish muscle (National Research Council, Canada).

|  | **INCT-CF-3 – Corn flour** | | | **DORM-2 – Dogfish muscle** | | |
| --- | --- | --- | --- | --- | --- | --- |
| Metal | Certified value | Measured value (n=3) | Recovery (%) | Certified value | Measured value (n=3) | Recovery (%) |
| Cr | 0.137 | 0.136±0.033 | 99 | Cr | 34.7±5.5 | 33.9±2.2 |
| Ni | 0.383±0.039 | 0.38±0.051 | 99 | Ni | 19.4±3.1 | 19.1±1.1 |
| Cu | 1.63±0.13 | 1.59±0.09 | 97 | Cu | 2.34±0.16 | 2.52±0.10 |
| As | 0.01 | 0.0096±0.001 | 96 | As | 18±1.1 | 17.7±1.6 |
| Cd | 0.007 | 0.0069±0.001 | 99 | Cd | 0.043 ± 0.008 | 0.044±0.004 |
| Pb | 0.052 | 0.051±0.003 | 98 | Pb | 0.065 ± 0.007 | 0.068±0.005 |
