# Supplementary material for: Carcinogenic and non-carcinogenic health hazards of potentially toxic elements in commonly consumed rice cultivars in Dhaka city, Bangladesh
Source: PLoS One. 2024 May 14;19(5):e0303305. doi: 10.1371/journal.pone.0303305 (PMC11093395; doi:10.1371/journal.pone.0303305)
Supplement: S2 Table — (DOCX) [file pone.0303305.s002.docx]

**S2 Table.** ICP-MS operating conditions and measurement parameters.

Operating conditions

Spray chamber Scott double-pass

Nebuliser pump (rps) 0.1

RF power (W) 1550

RF Matching (V) 0.2

Sample depth (mm) 8

Torch-H (mm) 0.2

Torch-V (mm) 0.2

Plasma gas flow rate (l min−1) 15

Carrier gas (Ar) flow rate) 0.9 (optimized daily)

(l min−1)

Measurement parameters

Scanning mode Peak hop

Resolution (amu) 0.7

Readings/replicate 1

Number of replicates 3

Isotopes ^75^As, ^111^Cd, ^208^Pb, ^52^Cr, ^60^Ni
