# Supplementary material for: Carcinogenic and non-carcinogenic health hazards of potentially toxic elements in commonly consumed rice cultivars in Dhaka city, Bangladesh
Source: PLoS One. 2024 May 14;19(5):e0303305. doi: 10.1371/journal.pone.0303305 (PMC11093395; doi:10.1371/journal.pone.0303305)
Supplement: S3 Table — (DOCX) [file pone.0303305.s003.docx]

**S3 Table.** Heavy metals concentration (mg/kg fw) in rice samples.

|  | | **Bashful** | **Katari** | **Lalbiroi** | **Basmati** | **Kaligira** | **BRRI-32** | **Naigarsail** | **Minikit** | **Pajam** | **Chinigura** |
| --- | --- | --- | --- | --- | --- | --- | --- | --- | --- | --- | --- |
| As | 1st digest | 0.05 | 0.12 | 0.16 | 0.19 | 0.35 | 0.11 | 0.19 | 0.09 | 0.19 | 0.04 |
|  | 2nd digest | 0.06 | 0.26 | 0.09 | 0.21 | 0.31 | 0.10 | 0.20 | 0.10 | 0.27 | 0.03 |
|  | 3rd digest | 0.05 | 0.26 | 0.10 | 0.34 | 0.39 | 0.31 | 0.24 | 0.30 | 0.35 | 0.04 |
| Cd | 1st digest | 0.06 | 0.09 | 0.01 | 0.16 | 0.04 | 0.05 | 0.01 | 0.11 | 0.02 | 0.10 |
|  | 2nd digest | 0.06 | 0.08 | 0.01 | 0.11 | 0.06 | 0.05 | 0.01 | 0.11 | 0.01 | 0.09 |
|  | 3rd digest | 0.05 | 0.09 | 0.01 | 0.11 | 0.06 | 0.05 | 0.02 | 0.22 | 0.09 | 0.10 |
| Pb | 1st digest | 0.04 | 1.24 | 0.82 | 0.18 | 0.02 | 0.06 | 0.03 | 0.01 | 0.01 | 0.06 |
|  | 2nd digest | 0.04 | 0.94 | 0.63 | 0.14 | 0.01 | 0.07 | 0.09 | 0.04 | 0.01 | 0.06 |
|  | 3rd digest | 0.04 | 1.05 | 0.51 | 0.14 | 0.01 | 0.07 | 0.08 | 0.41 | 0.01 | 0.07 |
| Zn | 1st digest | 10.97 | 12.96 | 11.38 | 16.05 | 15.97 | 33.79 | 20.75 | 22.25 | 20.57 | 12.31 |
|  | 2nd digest | 10.65 | 15.27 | 18.31 | 18.72 | 14.08 | 35.52 | 17.73 | 29.39 | 23.76 | 12.37 |
|  | 3rd digest | 10.59 | 15.25 | 20.19 | 20.23 | 16.37 | 33.74 | 21.02 | 27.06 | 22.33 | 12.37 |
| Cu | 1st digest | 7.96 | 5.42 | 2.58 | 12.67 | 15.24 | 5.41 | 7.28 | 4.91 | 8.42 | 6.55 |
|  | 2nd digest | 6.65 | 4.11 | 1.73 | 8.29 | 14.10 | 4.11 | 8.01 | 4.00 | 8.25 | 5.59 |
|  | 3rd digest | 6.37 | 5.25 | 1.64 | 8.71 | 10.92 | 4.84 | 8.78 | 4.14 | 8.23 | 6.15 |
| Cr | 1st digest | 0.93 | 0.92 | 0.24 | 1.81 | 1.70 | 0.76 | 0.84 | 0.69 | 0.96 | 1.41 |
|  | 2nd digest | 0.83 | 0.87 | 0.12 | 1.40 | 1.48 | 0.84 | 0.69 | 0.61 | 0.89 | 1.07 |
|  | 3rd digest | 0.98 | 0.90 | 0.19 | 1.05 | 1.08 | 0.73 | 0.62 | 0.64 | 0.68 | 1.29 |
| Mn | 1st digest | 6.98 | 5.03 | 10.97 | 3.48 | 11.37 | 27.83 | 11.34 | 2.56 | 4.36 | 2.57 |
|  | 2nd digest | 6.34 | 4.95 | 11.37 | 5.49 | 8.24 | 19.88 | 11.40 | 2.44 | 6.27 | 2.33 |
|  | 3rd digest | 7.65 | 3.98 | 13.71 | 3.81 | 8.20 | 18.52 | 11.37 | 2.62 | 5.30 | 2.63 |
| Ni | 1st digest | 7.01 | 0.95 | 0.39 | 3.08 | 1.26 | 0.49 | 0.60 | 0.93 | 0.52 | 0.52 |
|  | 2nd digest | 5.87 | 0.98 | 0.12 | 2.51 | 1.62 | 0.68 | 1.40 | 1.40 | 0.80 | 0.48 |
|  | 3rd digest | 5.01 | 0.80 | 0.13 | 1.52 | 1.63 | 0.67 | 1.25 | 1.92 | 0.46 | 0.53 |

fw: Fresh weight.
